# A phase II study investigating the re-induction of endocrine sensitivity following chemotherapy in androgen-independent prostate cancer

**DOI:** 10.1038/sj.bjc.6604051

**Published:** 2008-01-08

**Authors:** J Shamash, A Davies, W Ansell, S Mcfaul, P Wilson, T Oliver, T Powles

**Affiliations:** 1 Department of Medical Oncology, St Bartholomew's Hospital, London, UK

**Keywords:** re-induction, endocrine sensitivity, prostate cancer, chemotherapy

## Abstract

When chemotherapy is used in androgen-independent prostate cancer (AIPC), androgen deprivation is continued despite its failure. In this study, we investigated whether it was possible to re-induce hormone sensitivity in previously castrate patients by stopping endocrine therapy during chemotherapy. A phase II prospective study investigated the effects of reintroduction of endocrine therapy after oral chemotherapy in 56 patients with AIPC, which was given without concurrent androgen deprivation. After chemotherapy, patients were given maximum androgen blockade until failure when treatment was switched to diethylstilbestrol and dexamethasone. Patients had already received these endocrine treatments in the same sequence before chemotherapy. All patients were castrate at the start of chemotherapy. Forty-three subsequently restarted endocrine therapy after the completion of chemotherapy. The median overall survival for these 43 patients from the time of restarting endocrine therapy was 7.7 months (95% confidence interval (CI): 3.7–10.9 months). Sixteen (37%) patients had a 50% PSA response to treatment, which was associated with improved overall survival (14.0 months *vs* 3.7 months *P*=0.003). Eight out of 12 patients who did not respond to diethylstilbestrol before chemotherapy did so post chemotherapy. Re-induction of hormone sensitivity can occur after chemotherapy in AIPC.

There is an increasing number treatment options for androgen-independent prostate cancer (AIPC). Responses have been seen to steroids and estrogens (Farrugia *et al*, 2000; Nishimura *et al*, 2000), and chemotherapy has shown a survival advantage for these patients (Petrylak *et al*, 2004; Tannock *et al*, 2004). Until recently, it has been a universal practice to continue androgen deprivation during chemotherapy, despite previous progression on this treatment. Obviously, stopping treatment could result in restoration of androgen levels and growth of androgen-sensitive clones. However preclinical data suggest that chemotherapy in AIPC cell lines is more effective if given sequentially rather than concurrently (Tang *et al*, 2006). Additionally, in breast cancer, sequential hormone therapy and chemotherapy might be advantageous (Cavalli *et al*, 1983; Kim *et al*, 2005).

In a previous chemotherapy study involving castrate patients AIPC, we anecdotally observed that re-induction of hormone sensitivity was possible if endocrine therapy was stopped during chemotherapy (Shamash *et al*, 2005). Therefore, a prospective phase II study was designed to investigate this observation.

## MATERIALS AND METHODS

Between May 2003 and January 2006, castrate patients (testosterone <1.5 nmol l^−1^) with AIPC, who had in addition also failed diethylstilbestrol (1 mg) and dexamethasone (2 mg), were recruited into this study. All patients received oral chemotherapy with chlorambucil 1 mg kg^−1^ starting day 1, lomustine 2 mg kg^−1^ day 1, etoposide 50 mg daily from day 22–28 and 36–42, with the cycle repeating every 56 days. This treatment was based on a previously published regimen (Shamash *et al*, 2005). Patients were biochemically castrate (testosterone <1.5 nmol l^−1^ or failed maximum androgen blockade (MAB)) before starting the chemotherapy. All endocrine therapy was stopped during chemotherapy.

The entry criteria required for this study included histologic verification of prostate cancer, prostate-specific antigen (PSA) progression of disease despite failure of androgen deprivation and subsequent oestrogen (diethylstilbestrol 1 mg at least or equivalent) and corticosteroid (dexamethasone 2 mg or equivalent). This study was approved by the ethics committee and patients gave written informed consent before therapy.

Following failure of chemotherapy, patients recommenced endocrine therapy, initially with MAB (bicalutamide and GnRH analogue), and when this failed, patients were given dexamethasone 2 mg daily and diethylstilbestrol 1 mg daily (DS). Patients were seen monthly with PSA measurement, clinical and toxicity assessment.

In this study, progression of disease was based on PSA, using the Prostate-Specific Antigen Working Group criteria published in 1999 throughout (Bubley *et al*, 1999).

Thirteen of the 56 chemotherapy patients in this study did not restart endocrine therapy at the time of progression on chemotherapy. Twelve of these patients died of progression of disease before reintroduction of endocrine therapy could occur, and one refused to go back on endocrine therapy.

The log-rank test was used to look at which categorical variables were significant on univariate analysis for overall survival at the time of reintroduction of hormone therapy. Multivariate analysis was performed by fitting a Cox proportional hazard model to the data.

The Mann–Whitney two-sample statistic was used to compare median values between groups. Frequency tables and proportions were examined using Fisher's exact test.

## RESULTS

**Figure 1:**
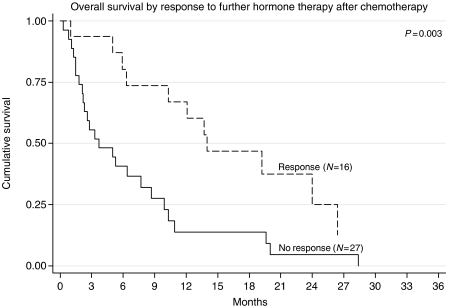
Kaplan–Meier curve comparing the outcome of patients with and without a 50% PSA response at reintroduction of hormone therapy (either MAB or diethylstilbestrol).

Forty-three patients with AIPC went on to receive further endocrine therapy after disease progression on chemotherapy. The characteristics of these patients are shown in Table 1, Figure 1.

The median overall survival for these patients, from the time of restarting endocrine therapy, was 7.7 months (95% CI: 3.7–10.9 months).

Twenty-two patients (51%) were non-castrate (testosterone >1.5 nmol l^−1^) at the start of endocrine therapy. Median overall survival for the non-castrate patients was 8.7 months (95% CI: 3.3–20.0) *vs* 6.4 months (95% CI: 2.6–12.1) for the castrate patients (*P*=0.33).

Sixteen of the 43 patients (37%) had a PSA response to treatment (either MAB or DS, which was associated with better overall survival compared with those with no PSA response (14.0 months (95% CI: 6.3–26.4) *vs* 3.7 months (95% CI: 2.1–8.7): *P*=0.003).

The median PSA progression free survival on MAB after chemotherapy was 1.7 months (range: 0.3–9.7 months). Eight (19%) patients had a PSA response to MAB, and these responders were on treatment for a median of 3.9 months (range 0.4–6.8). Two of 17 (12%) biochemically castrate patients had a PSA response to MAB, while six of 22 (27%) non-castrate patients had a PSA response to MAB (*P*=0.43).

Twenty-eight patients (65%) of the cohort went on to subsequently receive DS after MAB. The remainder did not receive DS because of contraindications to treatment or rapid disease progression on MAB. The median survival from the time of starting DS was 9.8 months (95% CI: 5.5–16.4). Twelve (43%) of these patients had a PSA response. Obtaining a PSA response to DS was associated with improved survival (median overall survival 16.5 months (95%CI: 6.9 to infinity) *vs* 5.5 months (95% CI: 2.7–13.3) (*P*=0.02). Overall, four patients responded to both MAB and DS.

The response to DS was independent of whether or not the patients were castrate at the end of chemotherapy (4/13 *vs* 8/14 (*P*=0.25)). Eight out of 12 patients who did not respond to DS before chemotherapy subsequently responded when rechallenged.

In multivariate analysis, the established prognostic factors (Gleason score, PSA at diagnosis, presence of bone metastasis) were unable to predict the likelihood of further hormone response following failure of chemotherapy.

## DISCUSSION

These prospective data confirm our anecdotal observation and suggest that endocrine sensitivity can be reintroduced by stopping endocrine therapy during chemotherapy for AIPC. Therefore, stopping endocrine therapy during chemotherapy may not be harmful, especially for the subset of patients who respond to it a second time. Randomised studies are required to answer this question formally.

It is intriguing that a group of patients should respond to endocrine therapy for a second time. This includes biochemically castrate and non-castrate patients, which suggests that when progression of disease occurs during chemotherapy, in the absence of concurrent androgen deprivation, both hormone-dependent and -independent clones may be responsible for the tumour regrowth. Therefore, it appears that chemotherapy may alter the subsequent behaviour of the disease. This theory is supported by the observation that some patients responded to DS after but not before chemotherapy. Additionally, patients who remained biochemically castrate after chemotherapy were less likely to respond to MAB than those who were not. Nevertheless, a large proportion of this group responded to DS, which was the treatment they received immediately before chemotherapy.

One of the possible confounding factors of this work is that patients responding to endocrine therapy a second time may have more indolent tumours and the PSA response is irrelevant. However, the PSA doubling time before chemotherapy did not correlate with a subsequent response to endocrine therapy (52 *vs* 57 days (*P*=0.60)).

There are a number of postulated mechanisms for the development of androgen independence in prostate cancer, including mutations of the androgen receptor to allowing other molecules to bind to it (Grossmann *et al*, 2001). There are *in vitro* data to suggest that this may be the case with diethylstilbestrol (Kalach *et al*, 2005). It may be that the prostate cancer cells, which regrow during chemotherapy in the presence of androgen ablation therapy, have molecular characteristics different from those for which chemotherapy is given alone. It is even possible that removing hormonal suppression during chemotherapy may allow cells to enter the cell cycle and become more sensitive to cytotoxic drugs.

A shortcoming of this work was that it did not use docetaxel chemotherapy, which has become the standard treatment for these patients. However, the case report published in this edition of the *British Journal of Cancer* suggests this may be possible with docetaxel and requires further investigation.

## Figures and Tables

**Table 1:** The characteristics of patients receiving endocrine therapy after chemotherapy in this study

Number	43
Median age	72 (range: 50–86)
Median PSA at the start of chemotherapy	144 (range: 2.1–981)
PSA at the time of chemotherapy progression	221 (11.5–3621)
Survival from time of androgen independence (months)	31.5 (95% CI: 20.3–43.5)
Time from androgen independence to starting chemotherapy (months)	14.6 (95% CI 10.5–19.0)
Duration on chemotherapy (months)	3.3 (2.5–4.6)
	
*Performance status at start of*	
0–2 months	29 (67%)
3 months	14 (33%)
	
Presence of metastatic disease	26 (76%)
	
*Gleason score*	
2–7	24
8–10	19
	
*Endocrine status at the end of chemotherapy:*	
Castrate	15 (35%)
Non-castrate	21 (49%)
Unknown	7 (16%)
	
Response to chemotherapy (50% PSA response)	8 (19%)
Median survival from the end of chemotherapy	7.0 (4.2–11)

CI=confidence interval; PSA=prostate-specific antigen.
